# Changes of CD4^+^ CD25^+^ Regulatory T Cells, FoxP3 in Adjuvant Arthritis Rats with Damage of Pulmonary Function and Effects of Tripterygium Glycosides Tablet

**DOI:** 10.1155/2012/348450

**Published:** 2012-12-27

**Authors:** Wan Lei, Liu Jian

**Affiliations:** ^1^Hubei University of Chinese Medicine, Wuhan, Hubei 430065, China; ^2^Department of Rheumatism Immunity, First Affiliated Hospital, Anhui College of Traditional Chinese Medicine, Hefei, Anhui 230031, China

## Abstract

*Objective*. To observe the effects of tripterygium glycosides tablet (TPT) on swelling degree, arthritis index (AI), pulmonary function, cytokines, the expression of regulatory T cells (Treg), and Foxp3 in rats of adjuvant arthritis. *Methods*. Rats were averagely divided into normal control (NC) group, model control (MC) group, methotrexate (MTX) group, and tripterygium glycosides tablet (TPT) group. Except for the rats of normal group, the others were intracutaneously injected with 0.1 mL of Freund's complete adjuvant in the right hindlimb. NC group and MC group were treated with physiological saline. MTX group and TPT group were treated with MTX, TPT, respectively. *Results*. The levels of swelling degree, AI, the alveolar inflammation integral, TNF alpha (TNF-*α*), and endothelium-1 (ET-1 ) in MC group were significantly increased (*P* < 0.01), and the levels of forced vital capacity (FVC), 25% vital capacity of the peak expiratory flow (FEF_25_), 50% vital capacity of the peak expiratory flow (FEF_50_), 75% vital capacity of the peak expiratory flow (FEF_75_), maximum midexpiratory flow (MMF), peak expiratory flow (PEF), interleukin-10 (IL-10), CD4^+^ CD25^+^ Treg, and Foxp3 were decreased (*P* < 0.01). The scores of alveolitis and ET-1 were decreased with treatment of TPT. The levels of FVC, FEF_25_, FEF_50_, FEF_75_, MMF, PEF, IL-10, and CD4^+^ CD25^+^ Treg in peripheral blood were increased. The expressions of Foxp3 protein and mRNA in lung tissue were also increased in TPT group. *Conclusions*. The paw swelling can be inhibited by TPT, and the inflammatory response in lung tissue was also decreased, which is a significant improvement in pulmonary function. The mechanism is probably associated with upregulating the expression of IL-10, Foxp3, and downregulating the level of TNF-*α*.

## 1. Introduction

Rheumatoid arthritis (RA) is an autoimmune arthritis affecting joints mainly, chronic inflammatory, along with many other tissues and organs. RA is an inflammatory disorder that principally attacks synovial joints. The process produces an inflammatory response of the synovitis secondary to hyperplasia of synovial cells, excess synovial fluid, and the development of pannus in the synovium. The pathology of the disease process often leads to the destruction of articular cartilage and ankylosis of the joints. Rheumatoid arthritis can also produce diffuse inflammation in the lungs. Owing to the lung tissue has redundant connective tissue and the close relation with blood vessels, and also has a link-intensive cycle system, the lung is one of the primary target organs. Interstitial lung disease (ILD) is the most common manifestation of rheumatoid lung disease [1–3]. About 0.8% of the world's population is afflicted by rheumatoid arthritis. In one study in Asia, the prevalence was 0.38% in the China region. Rheumatoid lung disease occurs in about 28% of patients, either in the beginning or during the course of their disease. The presence of rheumatoid lung disease is associated with severe active disease and increased mortality compared to the general population [4, 5]. Rheumatoid lung disease's etiology is unknown yet. Its pathogenesis may be related to CD4^+^ CD25^+^ regulatory T cells (Treg) (Figure 2) and Foxp3 expression in a previous study [6, 7].

Tripterygium glycosides tablet comprises triptolide which is a traditional medicinal plant that has been used in China for many years to treat inflammatory conditions including RA. In this study we will be testing tripterygium glycosides tablet that is superior to methotrexate in improving the paw swelling degree, arthritis index (AI), pulmonary function, and so on.

## 2. Methods

### 2.1. Experimental Animals

Rats were purchased from the Experimental Animal Center of Nanjing Medical University (Nanjing, China). All animals were housed under specific pathogen free (SPF) conditions and given free access to water and standard rat chow.

### 2.2. Drugs

1. Tripterygium glycosides tablet (TPT), 10 mg per piece, was produced by the Shanghai Medical Hongqi Pharmaceutical Factory, batch number NO. 20110819.
2. Methotrexate (MTX), 2.5 mg per piece, was produced by the Shanghai Traditional Chinese Medicine Co., Ltd. Xinyi Pharmaceutical Factory, batch number NO. 20111004.

### 2.3. Reagents

1. Elisa kit was purchased from R & D company, USA. Interleukin-10 (IL-10, Lot number 341225), Tumor Necrosis Factor alpha (TNF-*α*, Lot number NO. 341012). Endothelin-1 (ET-1, Lot number 340918).
2. Freund's complete adjuvant (FCA, Sigma, USA, Lot number NO. 098k8729).
3. Regulatory T cells kit: anti-mouse CD4-FITC (eBioscience, USA, Lot: 11-0040, Clone NO. OX35), anti-mouse CD25-PE (BioLegend, USA, Lot number: 202105, Clone NO. OX-39).
4. Anti-mouse Foxp3 monoclonal antibody (Santa Cruz, USA, Lot: SC-130666).
5. PCR Master Mix Kit and M-Mulv Reverse Kit (Fermentas, Canada, Lot: K0171, K1622).

### 2.4. Instruments and Equipment

1. Microplate reader was produced by Bio-TEK Corporation, USA (Model: ELX800).
2. PCR amplification was produced by Biometra Inc., Germany (Model: T1-Thermoblock, T-Gradient Thermoblock).
3. AniRes 2003 animal lung function analysis system was produced from Beijing Bei Lanbo Technology Co., Ltd., China.
4. Electrophoresis was produced by Amersham Corporation, USA (Model: EPS-301).

### 2.5. Model Copy, Grouping, and Administrations

The rats were randomly divided into four groups, the norma control (NC) group, model control (MC), methotrexate (MTX), and tripterygium glycosides tablet (TPT) group, 12 rats in each group. Except for the rats of NC group, the others were intracutaneously injected with 0.1 mL of Freund's complete adjuvant in the right hindlimb. Administration from 19th after inflammation, NC group and MC group were treated with physiological saline (1 mL/100 g per day). MTX group and TPT group were treated with MTX, 1 mL/100 g per week, and TPT, 1 mL/100 g per day.

### 2.6. Determination of the Experimental Index

#### 2.6.1. Paw Swelling and Arthritis Index

Paw swelling was measured in the right hindlimb of rats and calculated swelling degree [8]. 12th after inflammation, the joints were observed and recorded, once every three days. Calculated AI by Five scoring method [9]. no swelling (0 point); swelling from little toe joint (1 point); swelling from toe joints and foot (2 points); swelling from ankle and below (3 points); swelling from all of ankle (4 points).

#### 2.6.2. Paw Swelling Degree

We have

(1) $$\begin{array}{c} E\left(\%\right)=\frac{\left(V_{t}-V_{n}\right)}{V_{n}}\times 100\% \end{array}$$

(*V*_*n*_, *V*_*t*_ represent the volume before and after modeling).

#### 2.6.3. Lung Index

We have

(2) $$\begin{array}{c} \text{Lung}\;\;\text{index}\;\left(\text{LI}\right)=\frac{\text{lung}\;\;\text{wet}\;\;\text{weight}\;\left(\text{mg}\right)}{\text{body}\;\;\text{weight}\;\left(\text{g}\right)}\times 100\%. \end{array}$$

### 2.7. Morphology of Lung Tissue

30th after administration, remove the lungs tissue, and tissue was fixed in 4% paraformaldehyde 8 hr. Dehydration, transparent, dipping wax, embedding, slicing and HE staining successively. According to Szapiel method to determine the extent of alveolitis [10]: 0 points: Without alveolitis (−); 1 points: mild alveolitis (+), lesions were confined to below 20% of total lung tissue; 2 points: moderate alveolitis (++), lesions reach about 40%–50% of total lung tissue; 3 points: severe alveolitis (+++), lesions were more than 50 percent of total lung tissue. These results recorded 0, 1, 2, and 3 points, respectively.

### 2.8. Pulmonary Function

Pulmonary function parameters have forced vital capacity (FVC), 1 SEC. average expiratory flow (FEV1/FVC %), 25% vital capacity of the peak expiratory flow (FEF25), 50% vital capacity of the peak expiratory flow (FEF50), 75% vital capacity of the peak expiratory flow (FEF75), maximum mid-expiratory flow (MMF), peak expiratory flow (PEF). Process of the operation [11]: the rats were anesthetized with chloral hydrate (10%, 0.35 mL/100 g) through intraperitoneal injection and underwent tracheotomy endotracheal intubation. The rats were put inside the body of description to keep head low, and the ventilator tube was connected to mechanical ventilation in order to test pulmonary function. In the determination process, use the way that external pressure to make animal deep inspiration/deep breath. The computer measured each indicator automatically, quickly, and accurately.

### 2.9. Detection of CD4^+^ CD25^+^ Treg by Flow Cytometry

Whole blood samples were taken into K3-EDTA containing tubes and added to each tube with blood (10^6^ cells per tube), anti-mouse CD4-FITC (0.25 ug), anti-mouse CD25-PE (1.0 ug) successively. Keep them in dark place about 20–30 minutes at room temperature (20–25°C). Add 1 mL RBC lysate to tube and incubate for 15–25 minutes in dark again. Washing with PBA twice, centrifugation, the supernatant was given up. Detection of the expression of CD4^+^ CD25^+^ Treg was by flow cytometry [12].

### 2.10. Reverse Transcription-PCR

RNA was extracted by TRIZOL (TaKaRa Co., Japan) from 100 mg lung tissue and quantified using a spectrophotometer (Eppendorf Co., German). Three micrograms of total RNA were reverse transcribed into cDNA using Murine Moloney Leukemia virus (M-MLV) reverse transcriptase (Promega, USA). PCR was carried out according to the manufacturer's instructions. The house keeping gene GAPDH (GenBank accession: NM-017008): sense: 5′-TCC ACC ACC CTG TTG CTG TAG-3′, and antisense: 5′-CCA CAG TCC ATG CCA TCA CT-3′. amplified fragment 258 bp. The Foxp3 gene (GenBank accession: NM-001108250): sense: 5′-GCA AAC GGA GTC TGC AAG TG-3′, and antisense: 5′-GCA GGA GCT CTT GTC CAC TGA-3′, amplified fragment 450 bp. The targeted DNA amplified specifically was confirmed by electrophoresis and sequencing. PCR products were analyzed using Gel Works software after scanning the ethidium bromide-stained 1.5% agarose gel.

### 2.11. Western Blotting

Protein was lysed in gel-loading buffer containing 50 mM Tris-HCl (pH 6.8), 100 mM dithiothreitol, 2% sodium dodecyl sulfate, 0.1% bromophenol blue, and 10% glycerol. Fifty micrograms of total protein was resolved by SDS polyacrylamide gel electrophoresis and electrically blotted onto a nitrocellulose membrane. The filters were blocked with phosphate buffered saline (PBS) containing 15% nonfat milks. Detection of Foxp3 or beta Actin was carried out by western blot analysis with the mouse anti-Foxp3 monoclonal antibody (1 : 500) or the rabbit anti-beta Actin polyclonal antibody (1 : 5000) as the primary antibody, and goat anti-mouse or goat anti-rabbit IgG-conjugated horseradish peroxidase as secondary antibody. The bands were visualized by using the enhanced chemiluminescence system.

### 2.12. Enzyme-Linked Immunosorbent Assay (ELISA)

Levels of TNF-*α*, IL-10, and ET-1 in supernatants of different groups were measured using commercially available ELISA kits according to the test protocols. Values were expressedas pg/mL. The ELISA standard curve was prepared using a serial dilution of TNF-*α*, IL-10 and ET-1 standard protein concentrations. Absorbance was measured at 450 nm using a Multiskan-MK3 OD reader. The levels of recombinant TNF-*α*, IL-10, and ET-1 in the supernatant of the cells culture were calculated from the OD_450_ values according to the ELISA curve of the commercial TNF-*α*, IL-10, and ET-1 standards.

### 2.13. Immunohistochemistry

Lung tissue was dewaxed and rehydrated followed by antigen retrieval through microwaving in 2 mM EDTA (pH 9.0) for Foxp3 antigen. Sections were blocked with 5% bovine serum albumin (diluted in PBS) for 30 min and then incubated with each primary antibody in a moist chamber at 4°C overnight. Parallel sections from the same tissue block were used for the staining of all molecular variables. After washing in PBS, HRP polymer-linked secondary antibody was added for 60 min at room temperature. The sections were then visualized with DAB and counterstained with hematoxylin. Sections for the negative control were prepared using rabbit IgG1 or mouse IgG1 instead of primary antibody under the same experimental conditions.

### 2.14. Statistical Analyses

Continuous variables are the mean ± standard deviation. All samples were tested to ascertain if they followed a normal distribution. Data comparison among groups was carried out using ANOVA. Comparison between groups was carried out using the independent samples *t*-test. SPSS ver17.0 software was used for data analyses. *P* < 0.05 was considered significant.

## 3. Results

### 3.1. Effects of TPT on Paw Swelling Degree and Arthritis Index

Before inflammation, there was no obvious difference between paw swelling degree and arthritis index in each group. Before administration, the level of paw swelling degree and arthritis index of MC group, MTX group, TPT group were significantly higher than that in the NC group (*P* < 0.05 or *P* < 0.01).

After administration, compared to the NC group, the level of paw swelling degree and arthritis index of MC group were increased significantly (*P* < 0.01). Compared to the MC group, the level of swelling degree and arthritis index of MTX and TPT group were reduced significantly (*P* < 0.01). Compared to the MTX group, the level of swelling degree and arthritis index of TPT group were reduced significantly (*P* < 0.01). 

### 3.2. Effects of TPT on Pulmonary Function in AA Rats (See Table 1)

30th after administration, pulmonary function parameters such as FEF_50_, FEF_25_, FVC, FEF_75_, MMF, and PEF were significantly decreased, then FEV_1_/FVC, LI and score of alveolitis were increased in MC group. FVC, FEF_25_, FEF_50_, FEF_75_, MMF, and PEF were decreased and the score of alveolitis was increased with treatment of TPT.

### 3.3. Effects of TPT on TNF-*α*, IL-10, and ET-1 in AA Rats (See Table 2)

30th after administration, compared to the NC group, the concentrations of TNF-*α*, ET-1 in serum and ET-1 in lung tissue were significantly increased, and IL-10 in serum was decreased obviously in the MC group (*P* < 0.01). The concentrations of TNF-*α* and ET-1 in TPT group were significantly lower than those in MC group, and the concentrations of IL-10 were significantly higher than those in MC group (*P* < 0.01). The differences of TNF-*α*, ET-1 between TPT group and MTX group were statistically significant (*P* < 0.01).

### 3.4. Effects of TPT on Treg in Peripheral Blood, Foxp3 Expression in Lung Tissue

**Figure 1:**
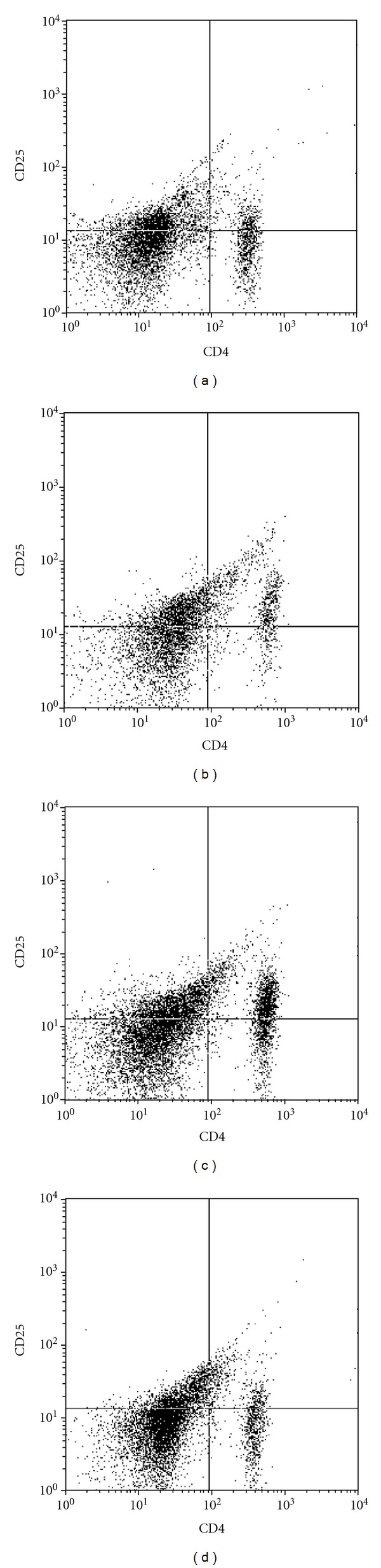
CD4^+^ CD25^+^ Treg changes of peripheral blood in rats (%) (a) MC group; (b) NC group; (c) TPT group; (d) MTX group.

**Figure 2:**
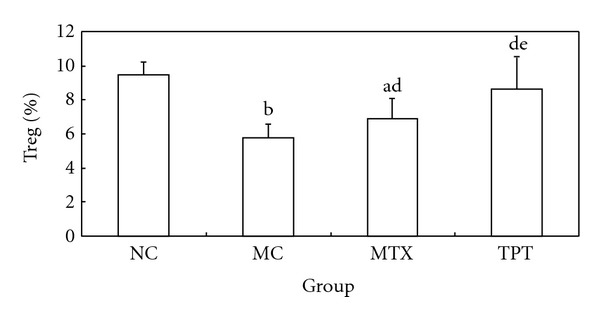
Comparisons of regulatory T cells in each group. Note: Compared with NC group, ^a^ *P* < 0.05, ^b^ *P* < 0.01; compared with MC group, ^c^ *P* < 0.05, ^d^ *P* < 0.01; compared with MTX group, ^e^ *P* < 0.05, ^f^ *P* < 0.01.

**Figure 3:**
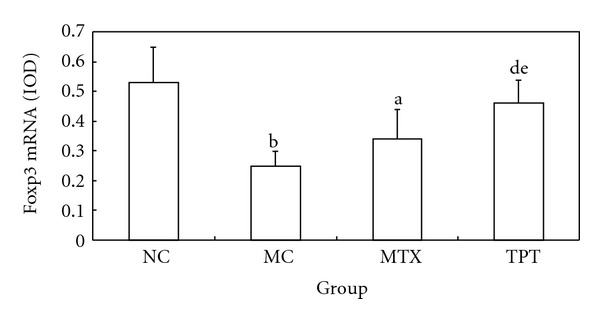
Comparisons of Foxp3 mRNA expression in each group. Note: compared with NC group, ^a^ *P* < 0.05, ^b^ *P* < 0.01; compared with MC group, ^c^ *P* < 0.05, ^d^ *P* < 0.01; compared with MTX group, ^e^ *P* < 0.05, ^f^ *P* < 0.01.

**Figure 4:**
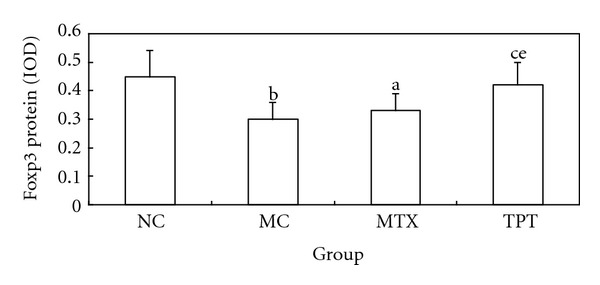
Comparisons of Foxp3 protein expression in each group. Note: compared with NC group, ^a^ *P* < 0.05, ^b^ *P* < 0.01; compared with MC group, ^c^ *P* < 0.05, ^d^ *P* < 0.01; compared with MTX group, ^e^ *P* < 0.05, ^f^ *P* < 0.01.

We detected Treg expression by flow cytometry in peripheral blood (Figures 1(a)–1(d)), and immunohistochemistry, RT-PCR, and western blot were used to detect the expression of Foxp3 in lung tissue. Flow cytometry results showed that the expression of CD4^+^ CD25^+^ Treg was decreased significantly in MC group and increased in TPT group. Immunohistochemistry results showed that the expression of Foxp3 in lung tissue was concentrated in nucleus and cytoplasm. The expression of Foxp3 was decreased in the MC group and increased in the TPT group. RT-PCR and western blot results showed that the expressions of Foxp3 mRNA and protein were decreased significantly in MC group (Figures 3 and 4).

## 4. Discussion

We detected that paw swelling, AI, alveolitis points, TNF*α*, and ET-1 expression were significantly increased in rats experimentally developed with adjuvant arthritis, while FVC, FEF_25_, FEF_50_, FEF_75_, MMF, PEF, IL-10, CD4^ +^ Treg, CD4^+^ CD25^+^ Treg, and Foxp3 expression in lung tissue were significantly decreased. These studies have shown inflammatory reaction was emerged in the joints of rats developed with AA, while their pulmonary function has been changed, the level of pulmonary function declines as a result. Pulmonary function change was further indicated by the observation of pulmonary ventilation function disorder, particularly restrictive ventilatory disorder, accompanied by small airway obstruction. It is worthwhile to note that an imbalance between proinflammatory and anti-inflammatory cytokine existed, suggesting that the inflammation was occurred in both joints and lung. The abnormal expression of regulate T cells and Foxp3 shows Treg was probably involved in RA pathogenesis of lung injury [13–17].

Modern pharmacological studies show that triptolide has anti-inflammatory and immunomodulatory active ingredients effects in experimental animals. Pharmacological and clinical experiments also show that the major effective component of tripterygium is alkaloids, may directly lead to the results that reduce capillary permeability, inhibit infiltration of inflammatory exudation, inhibit or counter modulate various types of inflammatory mediators as well as the anticoagulant, antiembolism, and reduce the damage on lung tissue [18, 19]. The immune modulatory effect of tripterygium ranged widely from the humoral immunity, cellular immunity, to macrophage phagocytosis. Triptolide, its monomer, has inhibition on inflammatory joint edema was induced by carrageenan, croton oil, and Freund's complete adjuvant. A study also showed that triptolide has improved the role that could improve the recovery of alveolitis and fibrosis. Triptolide can reduce the size of alveolitis and pulmonary fibrosis and increase alveolar space. In our results, compared with the AA rats, FVC, FEF_25_, FEF_50_, FEF_75_, MMF, PEF, IL-10, and the expression of regulate T cells, Foxp3 was significantly increased in the triptolide-treated group, while paw swelling, AI, TNF-*α*, and ET-1 decreased. There also found that the treatment effect of tripterygium was more obvious than that of triptolide.

The levels of CD4^+^ CD25^+^ Treg decreased in peripheral blood of the AA rats obviously. It may be because of that the diminished CD4^+^ CD25^+^ Treg level is correlated to the breakdown of the autoimmune balance and the development of rheumatoid arthritis-induced lung injury, for low level of CD4^+^ CD25^+^ Treg cannot sufficiently convert CD4^+^ CD25^−^ T cells into regulatory cells through immune induction. After the intervention of tripterygium, CD4^+^ CD25^+^ Treg was upregulated. We proposed that as a consequence, the CD4^+^ CD25^−^ T cells can be converted into Treg that secretes IL-10 in peripheral blood of the AA rats, thus promoting the expression of Foxp3 in lung tissue, then making the high level expression of anti-inflammation cytokine and the low level expression of proinflammatory cytokines. Finally the immunosuppression activity of CD4^+^ CD25^+^ Treg is exemplified exclusively, as our data implied.

Our results showed that tripterygium can obviously improve pulmonary function in AA rats, and the mechanism may function through inhibiting the expression of TNF-*α*, ET-1, upregulating CD4^+^ CD25^+^ Treg level, and promoting the expression of IL-10 and Foxp3 [20–26]. It is suggested that tripterygium can upregulate CD4^+^ CD25^−^ Treg and Foxp3 expression, playing the role of the immune adjustment [27–30]. TNF-*α* is a upstream regulatory factor of this process, which can induce accumulation of macrophages, secrete large amount of TGF-*β* and ET-1, expand the inflammatory response, and promote the formation of synovitis inflammation and lung disease. TNF-*α* not only induces the amplification of Th_2_ cells, but also limits the immunosuppression activity of CD4^+^ CD25^+^ regulatory T cells, which suppress immune tolerance [31–34]. In this study, we concluded that tripterygium plays a vital role of immune adjustment in lung injury.

## Figures and Tables

**Table 1:** Comparisons of pulmonary function, pulmonary coefficient, and alveolitis in rats ($n=12,\bar{x}\pm s$).

Index	Group			
	NC	MC	MTX	TPT
Pulmonary function				
FVC (mL)	6.09 ± 2.01	5.07 ± 0.27^b^	5.25 ± 0.25^b^	5.89 ± 0.30^ce^
FEF_25_ (mL/s)	45.2 ± 6.21	30.7 ± 2.81^b^	36.8 ± 3.67^bc^	42.2 ± 4.71^cd^
FEF_50_ (mL/s)	39.4 ± 6.84	27.0 ± 6.07^b^	30.3 ± 4.94^b^	40.2 ± 4.36^ce^
FEF_75_ (mL/s)	37.7 ± 5.87	37.7 ± 5.87	24.1 ± 5.80^b^	34.4 ± 10.5^cd^
MMF (mL/s)	39.2 ± 5.72	39.2 ± 5.72	31.7 ± 4.36^b^	37.1 ± 4.62^cd^
PEF (mL/s)	39.1 ± 4.87	39.1 ± 4.87	33.1 ± 3.12^b^	37.8 ± 4.33^cd^
Pulmonary coefficient	2.61 ± 0.08	0.36 ± 0.36^b^	2.83 ± 0.11^bc^	2.74 ± 0.12^bcd^
Alveolitis points (point)	0.37 ± 0.74	2.75 ± 0.70^b^	1.87 ± 0.35^bc^	1.62 ± 0.51^bcd^

Note: compared with NC group, ^a^ *P* < 0.05, ^b^ *P* < 0.01; compared with MC group, ^c^ *P* < 0.01; compared with MTX group, ^d^ *P* < 0.05, ^e^ *P* < 0.01.

**Table 2:** Comparisons of regulatory T cells, cytokines, and Foxp3 in rats ($\bar{x}\pm s$).

Index		Group			
	Case	NC	MC	MTX	TPT
CD4^+^CD25^+^Treg (%)	10	8.64 ± 1.88	5.78 ± 0.85^b^	5.92 ± 1.36^a^	7.96 ± 2.31^ce^

Cytokine in serum					
TNF-*α* (pg/mL)	10	32.1 ± 7.40	80.5 ± 10.7^b^	59.8 ± 20.3^bc^	53.2 ± 19.7^bd^
IL-10 (pg/mL)	10	106.6 ± 14.7	51.5 ± 20.1^b^	74.2 ± 23.6^bc^	90.2 ± 21.8^de^
ET-1 (pg/mL)	10	19.0 ± 8.11	48.3 ± 16.8^b^	32.2 ± 10.7^bc^	30.8 ± 12.6^ac^

Cytokine in lung tissue					
ET-1 (pg/mL)	10	8.03 ± 5.76	27.2 ± 15.7^a^	20.2 ± 11.9^a^	11.7 ± 9.33^cf^

Note: compared with NC group. ^a^ *P* < 0.05, ^b^ *P* < 0.01; compared with MC group. ^c^ *P* < 0.05, ^d^ *P* < 0.01; compared with MTX group, ^e^ *P* < 0.05, ^f^ *P* < 0.01.
